# High-Efficiency, Solution-Processed, Multilayer Phosphorescent Organic Light-Emitting Diodes with a Copper Thiocyanate Hole-Injection/Hole-Transport Layer

**DOI:** 10.1002/adma.201403914

**Published:** 2014-11-10

**Authors:** Ajay Perumal, Hendrik Faber, Nir Yaacobi-Gross, Pichaya Pattanasattayavong, Claire Burgess, Shrawan Jha, Martyn A McLachlan, Paul N Stavrinou, Thomas D Anthopoulos, Donal D C Bradley

**Affiliations:** Department of Physics and Centre for Plastic Electronics, Blackett Laboratory, Imperial College LondonLondon, SW7 2AZ, UK E-mail: a.perumal@imperial.ac.uk; t.anthopoulos@imperial.ac.uk; d.bradley@imperial.ac.uk; Department of Materials and Centre for Plastic Electronics, Imperial College LondonLondon, SW7 2AZ, UK

Following early breakthrough reports on vacuum-deposited small molecule and solution-processed conjugated polymer organic light-emitting diodes (OLEDs),[1] tremendous progress has been made in commercializing smartphone, tablet, and television display products. OLED lighting offers additional challenges including very demanding efficiency requirements set by the ≈100 lm W^–1^ luminous power efficiency of fluorescent lamps. Vacuum-processed OLEDs have recently passed this 100 lm W^–1^ target,[2] thereby stimulating continued interest in large area lighting applications. However, vacuum processing at scale, especially where shadow-masked pixellation is required, remains challenging and costly. Strong and growing interest has consequently been shown in solution-based processes (e.g. ink-jet[3a] or gravure printing[3b-d] or slot-die coating[3e]) to address these limitations and achieve the ultimate potential of plastic electronics in large-area, low-cost, high-throughput device fabrication.

Realizing solution-processed multilayer OLEDs with efficiency comparable to vacuum-deposited devices remains extremely challenging,[4] and **Table** **1** summarizes the performance of a selection of state-of-the-art devices fabricated using different approaches. We focus here on introducing a new solution-processed hole-injection/hole-transport layer (HIL/HTL) material, namely copper thiocyanate (CuSCN), and compare the performance of OLEDs using CuSCN with standard poly(3,4-ethylenedioxythiophene:polystyrenesulpho­nate) (PEDOT:PSS) HIL/HTL devices.

**Table 1:** Device characteristics for selected high-performance solution-processed OLEDs. The (PPy)_2_Ir(acac) results are taken from the present work

Emission material	Voltage [V] @ 1 cd m^–2^	Luminous efficiency [cd A^–1^] @1000 cd m^–2^	Luminous power efficiency [lm W^–1^] @1000 cd m^–2^	Approach
TEGna)	2.6	55	49	Cross-linking [4c] (2007)
Ir(mppy)_3_b)	2.8	50	30	EML comprising a four-component small molecule and polymer blend [4d] (2011)
Ir-based poly(dendrimer)	2.8	–	(32 @100 cd m^–2^)	Dendrimer [4g] (2012)
(2-CF_3_BNO)_2_Ir(acac)c)	–	94.5	69.3	Small molecule [4a] (2013)
(PPy)_2_Ir(acac)	2.7	47 (maximum value = 51)	22 (maximum value = 55)	EML comprising a three-component small molecule blend (2014)

a) *fac*-tris[2-((3-*p*-xylyl)phenyl)pyridine]iridium(III);

b) tris[2-(p-tolyl)pyridine]iridium(III);

c) bis[5-methyl-8-trifluoromethyl-5H-benzo(c)(1,5)naphthyridin-6-one]iridium(III)­(acetylacetonate).

HIL/HTL materials should ideally combine the following properties: (i) good adhesion to and planarization of the anode, (ii) high optical transparency across the full visible spectrum, (iii) a suitable work function to allow ready injection of holes from typical anode materials and transfer of those holes into the emission layer (EML), (iv) sufficient conductivity to allow low turn-on and operating voltages, (v) good electron-blocking properties to prevent leakage of electrons from the EML, and (vi) good exciton-blocking properties to confine the emissive states within the EML.[5] PEDOT:PSS has been the archetypical HIL/HTL material for solution-processed OLEDs and combines good conductivity with reasonable transparency and work function.[6] As with vacuum-deposited high-efficiency OLED device HIL/HTL materials,[5c] the conductivity of the PEDOT:PSS is achieved by oxidative doping. In the PEDOT:PSS system, PSS chains template the oxidative polymerization of ethylenedioxythiophene (EDOT) and are retained as a counter-ion scaffold that the doped (oxidized) PEDOT decorates and that promotes solubility and stability.[7a,b] PEDOT:PSS does, however, have several limitations, specifically that its acidity can damage indium tin oxide (ITO) anodes and EML materials, its work function is rather low, it is not very effective as an electron-blocking layer, and it does not have high thermal stability, especially in air.[7c,d] Insertion of arylamine polymer based thin film interlayers helps to address some of these issues[8] but unfavorably adds two process steps (interlayer coating and high temperature annealing) to device fabrication.

An alternative approach is to use an inorganic metal oxide HIL/HTL such as tungsten oxide (WO_3_), molybdenum oxide (MoO_3_), or nickel oxide (NiO).[4i,^9^] These are traditionally deposited via thermal evaporation under high vacuum[10] but solution-processed metal oxide layers are being explored, based on thermal decomposition of organic–inorganic hybrid precursors or on the deposition and annealing of suspensions of nanoparticles sheathed in organic solubilizing/stabilizing layers.[11] After coating, the metal oxide film normally requires continuous or flash thermal annealing at >300 °C or equivalent laser sintering to reach a moderate conductivity and transparency, processing steps that can be detrimental, especially for plastic substrates.[11d] Metal oxide films can also be relatively resistive restricting their use to that of thin (approximately few nanometer thick) HILs that may then require an additional, thicker, HTL on top, again increasing the fabrication step count.[11,12]

It should be evident from the preceding that there is ample scope to introduce new HIL/HTL materials that better satisfy the requirements listed above. Here, we focus on CuSCN, a p-type semiconductor that is transparent across the whole of the visible spectrum, only absorbing light at energies above 3.8 eV (≈325 nm). CuSCN is abundantly available at low cost from a variety of commercial sources and can be straightforwardly solution processed in air without needing a postdeposition thermal anneal. We report the use of CuSCN as an HIL/HTL for solution-processed OLEDs. To our knowledge, this is the first such report in the literature, although CuSCN has been used as the active semiconductor in thin film transistors[14] and as a HTL in organic bulk heterojunction solar cells.[15] The OLED devices we report with CuSCN as HIL/HTL perform significantly better than equivalent devices fabricated with a PEDOT:PSS HIL/HTL and we are able to demonstrate solution-processed, phosphorescent, small-molecule, green (≈525 nm peak wavelength) OLEDs with luminance ≥10 000 cd m^–2^, maximum luminous power efficiency ≤55 lm W^–1^, and maximum luminous efficiency ≈50 cd A^–1^.

**Figure** **1**a–c shows, respectively, a schematic diagram of our bottom anode, bottom-emitting OLED device architecture, the corresponding energy-level diagram for the component materials, and their chemical structures. The device structure is simple relative to state-of-the-art vacuum deposited OLEDs[2,16] with only three layers [HIL/HTL, EML, and electron transport layer (ETL)] inserted between the anode and cathode. Two device types were fabricated with ≈45-nm thick spin-coated PEDOT:PSS or CuSCN as HIL/HTL. The EML used for both device types comprised a guest-host blend with 5 wt% of the guest phosphorescent green emitter Bis(2-phenylpyridine)(acetylacetonate)iridium(III) ((PPy)_2_Ir(acac)) dispersed in a 60:40 volume blend of 2,6-Bis(3-(9H-carbazol-9-yl)phenyl)pyridine (26DCzPPy) and 4,4′,4′′-tris(*N*-carbazolyl)­triphenylamine (TCTA) as host. It was spin coated to a thickness of ≈50 nm. The low concentration of (PPy)_2_Ir(acac) emitter molecules in the EML avoids aggregation of emitter molecules and consequent exciton quenching.[17,18] Complete energy transfer from the 26DCzPPy:TCTA host to the (PPy)_2_Ir(acac) guest is achieved by means of a combination of Förster and Dexter transfer.[18,19] The EML materials were chosen such that the energy-level alignment of the host and the guest also energetically favor good charge-carrier injection and charge balance.[20–22] The high-lying triplet levels of the host molecules (≈2.71 eV for 26DCzPPy and ≈2.76 eV for TCTA)[20] relative to the (PPy)_2_Ir(acac) guest emitter (≈2.3 eV)[22] ensures that excitons should be confined to the guest. The 4,6-Bis(3,5-di(pyridin-3-yl)phenyl)-2-methylpyrimidine (B3PYMPM) ETL acts in the same way with a triplet energy (≈2.8 eV).[21]

**Figure 1:**
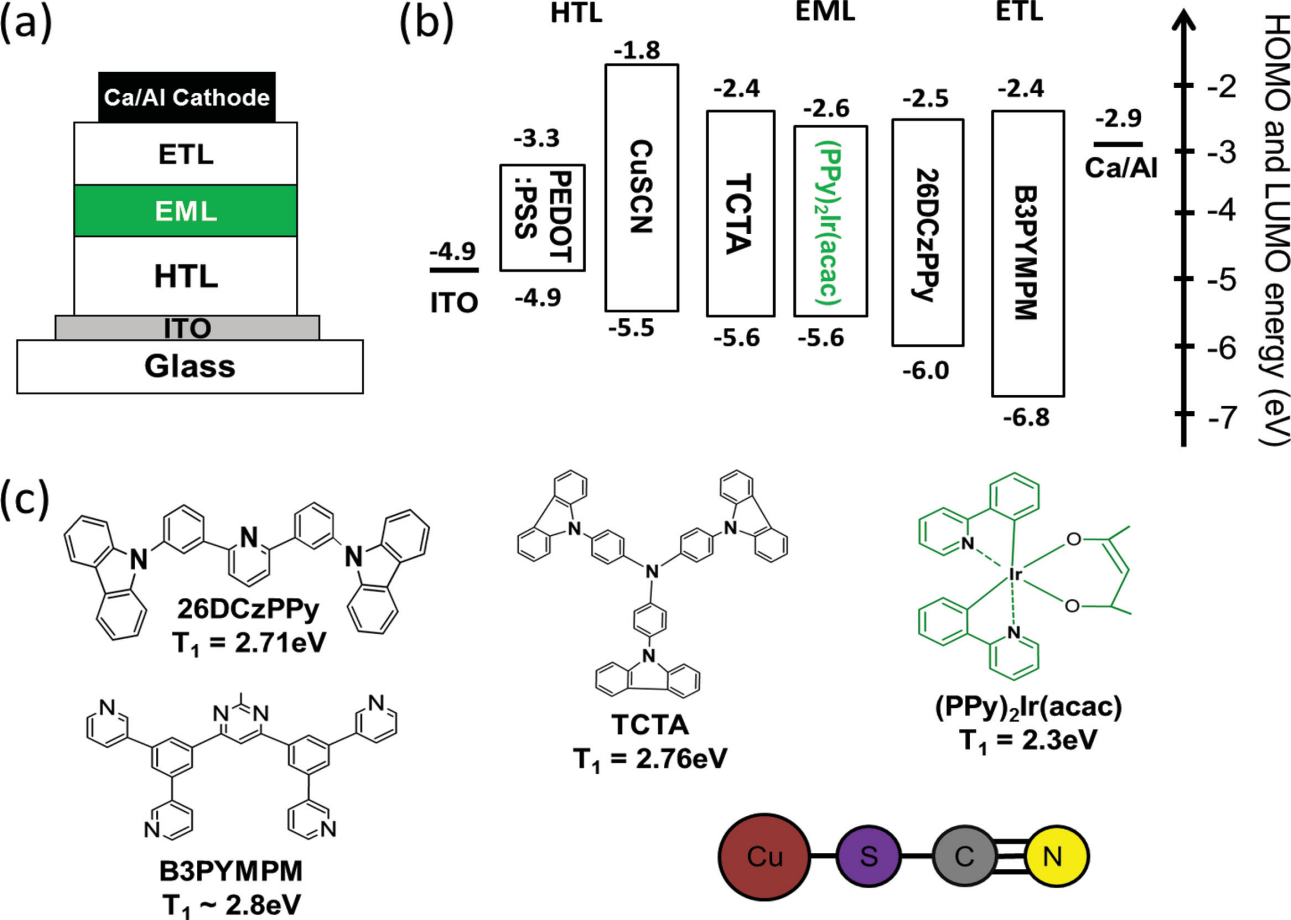
a) Schematic device structures for bottom-emitting OLEDs with PEDOT:PSS and CuSCN HIL/HTLs. b) Schematic energy level diagram for the component materials and c) their chemical structures and triplet energies (T_1_). The EML consists of 26DCzPPy:TCTA doped with (PPy)_2_Ir(acac). The HOMO, LUMO, and triplet energy levels are literature values.[20–22]

In the fabrication of multilayer OLEDs from solution, it is important that the solvent for each successive layer wets but does not dissolve/swell the previously deposited film(s); such solvents are described as orthogonal. Here, we spin coat an HIL/HTL on top of the insoluble ITO anode, spin coat the EML, and finally evaporate the ETL and Ca/Al cathode on top (cf. Experimental Section). The EML is dissolved in chlorobenzene (CB), which is known to be orthogonal to the standard water-soluble PEDOT:PSS HIL/HTL, thereby allowing straightforward device fabrication.[23] The CuSCN HIL/HTL is dissolved in diethyl sulfide (DES) and we have investigated the film formation of successive layers in CuSCN-based devices via cross-section transmission electron microscopy (TEM). **Figure** **2** shows a cross-section TEM image of the complete device, with many of the individual layers in the stack clearly resolved and identified. The EML and ETL are not resolved as there is insufficient scattering contrast between them due to their very similar chemical composition. The CuSCN HIL/HTL fills the indentations in the ITO anode and is in turn uniformly coated by the EML. The CuSCN/EML interface is sharp and continuous with no apparent gaps or voids, demonstrating that CB is orthogonal to the DES spin-coated CuSCN film. The combined EML and ETL organic layers further planarize the device structure and yield a very flat surface onto which the Ca and Al are deposited. This is consistent with AFM measurements on EML films spin coated on both quartz and CuSCN-coated quartz substrates that show a very smooth and homogeneous surface topography with a root mean square (RMS) roughness ≤ 0.5 nm (Figures S1 and S2, Supporting Information). The TEM thickness values are consistent with the thicknesses measured by surface profilometry for individual layers deposited under the same conditions on glass substrates.

**Figure 2:**
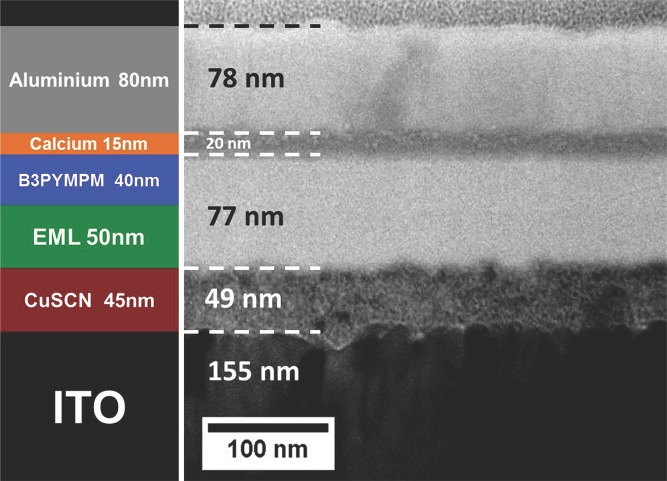
TEM image of the focused ion beam (FIB)-milled cross section of a complete CuSCN HIL/HTL OLED device (right side), together with a schematic of its stack structure (left side). The EML and ETL layers remain unresolved but the interface between HIL/HTL and EML is observed to be sharp and continuous, as indeed is the higher lying interface between ETL and Ca.

The, as-purchased, CuSCN powder has an X-ray diffraction pattern dominated by spacings characteristic of the orthorhombic *α-*phase polymorph (data not shown). Conversely, CuSCN thin film samples spin coated from DES solution have selective area electron diffraction patterns indicative of both the *α-* and hexagonal *β-*phase structures.[14b] This observation is consistent with reports in the literature[13] and suggests that further work to control and optimize the film microstructure for OLED performance would be of interest. Surface topographies for ITO, ITO/PEDOT:PSS, and ITO/CuSCN samples are shown in **Figure** **3**. The ITO film comprises polycrystalline grains with ≈100s nm dimensions ( Figure 3a) and has a RMS roughness of ≈4 nm. Covering the ITO with a PEDOT:PSS HIL/HTL ( Figure 3b) reduces the RMS roughness to ≈2 nm and fills in most of the voids. For ITO coated with a CuSCN HIL/HTL ( Figure 3c), the RMS roughness increases to ≈5 nm with ≈10 nm CuSCN crystalline grains covering the surface. UPS measurements on CuSCN films deposited in our laboratory have been reported elsewhere,[14] with the ionization potential, *I*_p_ ≈ 5.5 eV.

**Figure 3:**
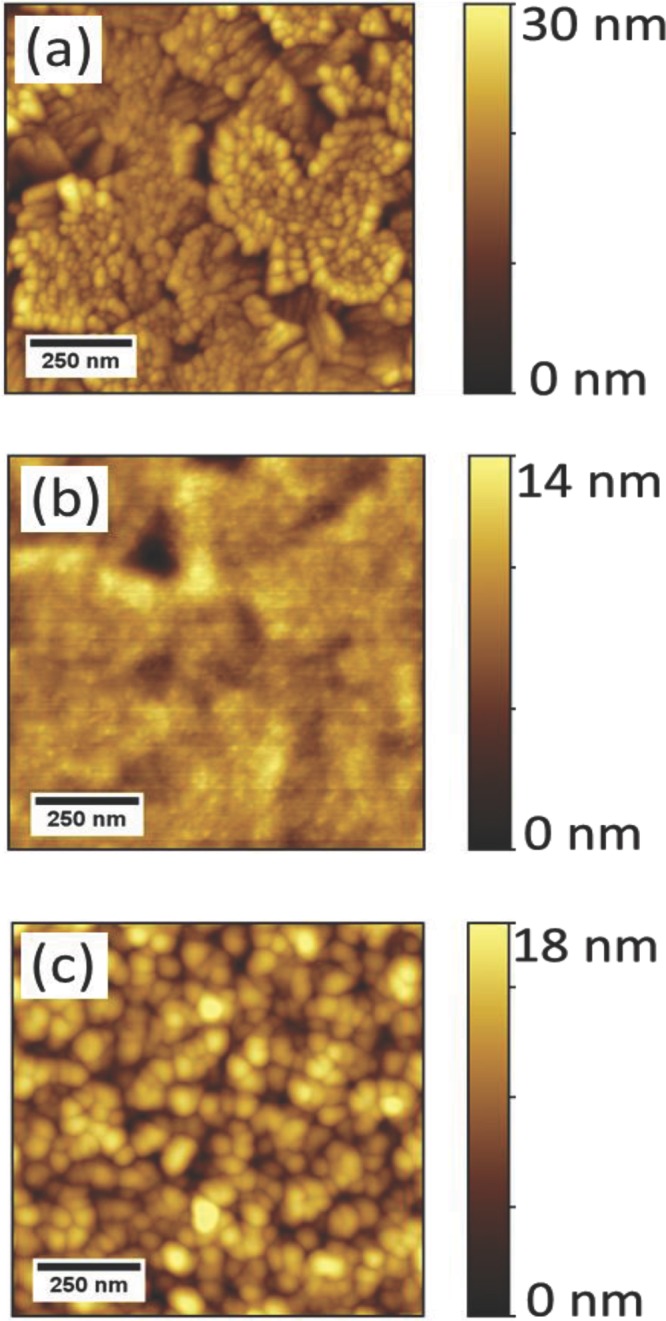
AFM topography images of a) ITO, b) ITO/PEDOT:PSS, and c) ITO/CuSCN.

The 250–1000 nm optical absorbance (*A*) spectra of 45-nm thick PEDOT:PSS (blue line) and CuSCN (red line) films deposited on quartz substrates are shown in **Figure** **4**, as deduced from the measured transmittance (*T*) and reflectance (*R*) spectra using *A* (%) = 100 – *R* (%) – *T* (%). CuSCN films absorb strongly below ≈325 nm with an exciton peak at ≈300 nm. Advantageously, however, they are weakly absorbing across the whole of the visible spectrum and into the near infrared (NIR). Conversely, PEDOT:PSS films absorb less strongly in the UV but have increasing absorption beyond ≈500 nm through the red and into the NIR. This arises from optical transitions among the intragap polaron levels that result from the oxidative doping that gives PEDOT:PSS its conductivity. For the (PPy)_2_Ir(acac) emitter used here (with emission peaked in the green (cf. **Figure** **5**a)), such absorption differences are largely immaterial. For organic solar cells, however, they can have a significant effect on short-circuit current density.[15] Diffuse reflectance measurements (Figure S3, Supporting Information) show that spin-coated CuSCN films are significantly scattering and that the scattering amplitude increases as the granularity grows (in thicker films). Scattering is of significant interest for device structures and can be advantageous to OLED light extraction[24] and organic photovoltaic (OPV) light trapping,[25] resulting in higher efficiencies; it also supports stimulated emission.[26] Again, it will be of interest to systematically adjust deposition conditions to control this effect.

**Figure 4:**
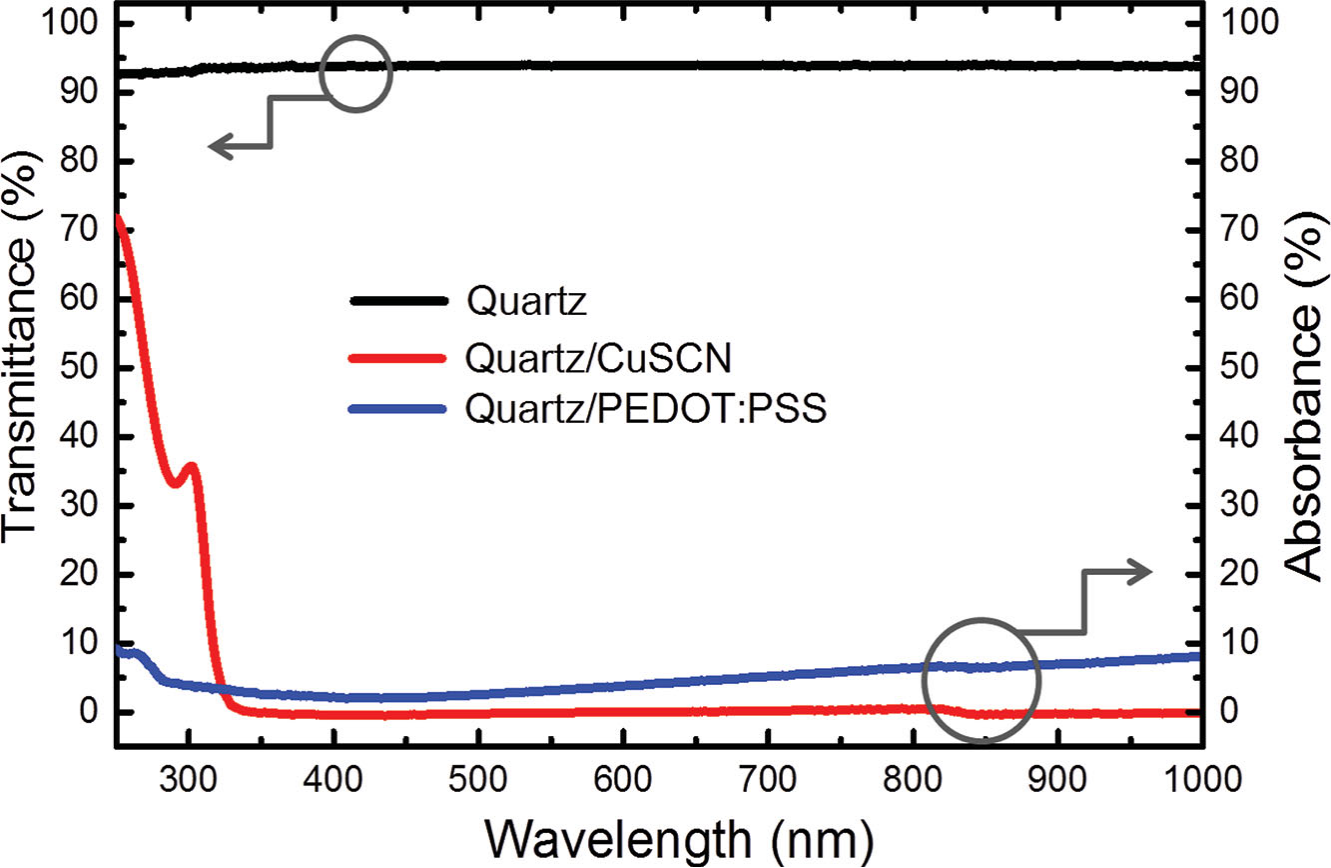
Optical absorbance spectra (right ordinate) for ≈45-nm thick PEDOT:PSS and CuSCN films spin coated on quartz substrates. Also shown for reference is the corresponding transmittance spectrum (left ordinate) recorded for a quartz substrate on its own.

**Figure 5:**
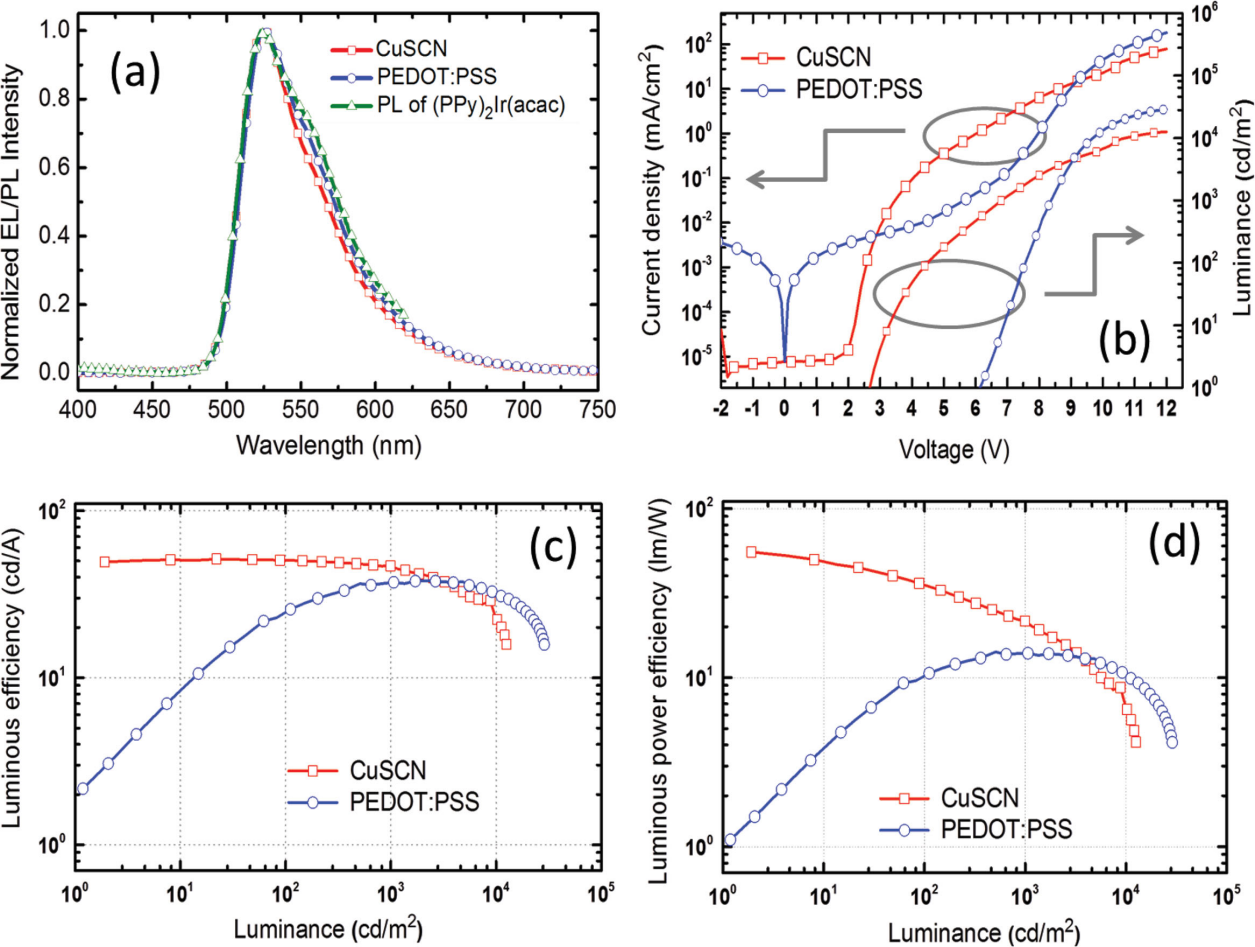
Comparison of OLED characteristics for CuSCN and PEDOT:PSS HIL/HTL structures: a) EL spectra for (PPy)_2_Ir(acac) emission from structures (cf. Figure 1a) with either PEDOT:PSS or CuSCN as HIL/HTL and recorded at 0.1 mA. The photoluminescence spectrum of (PPy)_2_Ir(acac) is also shown for reference. b) Current density/luminance versus voltage (J–V–L) characteristics for PEDOT:PSS (circles) and CuSCN (squares) structures. c) Luminous efficiency (cd A^–1^) versus luminance (cd m^–2^) characteristics for PEDOT:PSS (circles) and CuSCN (squares) structures. d) Luminous power efficiency (lm W^–1^) versus luminance (cd m^–2^) characteristics for PEDOT:PSS (circles) and CuSCN (squares) structures.

Electroluminescence (EL) spectra recorded at 0.1 mA are shown in Figure 5a together with the photoluminescence (PL) emission spectrum recorded under 320 nm optical excitation for a 50-nm thick (PPy)_2_Ir(acac) film spin coated on a quartz substrate. The EL closely matches the PL of (PPy)_2_Ir(acac) peaking at 523 nm for CuSCN and 524 nm for PEDOT:PSS devices; the PL peaks at 523 nm. The small spectral differences at wavelengths >540 nm are likely to be the result of photonic effects due to the different optical environments that the emitting molecules find themselves in; the changes are not due to differences in HIL/HTL absorption (cf. Figure 4). The EL peak did not shift appreciably with increasing voltage/current suggesting that the recombination zone location is relatively stable.

Figure 5b shows typical current density (left ordinate)/luminance (right ordinate) versus voltage (J–V–L) characteristics for CuSCN (squares) and PEDOT:PSS (circles) HIL/HTL OLEDs. The work function for PEDOT:PSS is reported to be 4.9–5.2 eV.[27] The highest occupied molecular orbital (HOMO) of TCTA is located at 5.6 eV[20] leading to an energy barrier of ≈0.4–0.7 eV for hole injection from PEDOT:PSS, significantly higher than would normally allow an ohmic contact. The high turn-on voltage ≈6 V (to reach 1 cd m^–2^ luminance) is consistent with such a barrier and further implies that direct injection into the (PPy)_2_Ir(acac) component of the EML blend does not occur to any significant degree. Another feature of the J–V data for the PEDOT:PSS HIL/HTL devices is the significant current density at low bias voltages in both forward and reverse direction, suggesting the presence of a number of leakage shunt paths.

On the other hand, for devices with a CuSCN HIL/HTL (valence band ≈5.5 eV below vacuum,[14] there is a substantially lower injection barrier (≈0.1 eV) to the HOMO of TCTA and the device consequently turns on at ≈2.7 V [reasonably close to the photon energy of the EL peak (523 nm = 2.37 eV)]. The presence of a beneficial interface dipole[28] at the ITO/CuSCN interface may also play a role. Another feature of CuSCN is that its conduction band is located at ≈2.0 eV[14] and therefore the HIL/HTL acts as an efficient electron-blocking layer. This is confirmed by a more than 100-fold lower leakage current density than for PEDOT:PSS, which has an electron affinity of ≈3.3 eV.[27] As a consequence, as already noted above, PEDOT:PSS HIL/HTL devices often employ a wide optical gap arylamine interlayer to address both the electron-blocking and hole-injection issues.[8] This appears not to be required for CuSCN devices, thereby saving additional deposition and annealing steps. Combined with the deep HOMO level of the B3PYMPM ETL that efficiently blocks holes, the high-lying conduction band of CuSCN also helps to tightly confine excitons within the EML.

At ≈9 V, the CuSCN and PEDOT:PSS J–V (and L–V) curves cross, signaling that for high bias voltages the PEDOT:PSS HIL/HTL sustains a higher device current density (and correspondingly enhanced luminance). Figure 5c,d provides further insight by comparing the luminous efficiency and luminous power efficiency (parametric in luminance) for both device types, and **Table** **2** summarizes the important performance indicators extracted from these and Figure 5b. PEDOT:PSS HIL/HTL devices initially have low efficiencies that steadily improve with increasing luminance up to ≈1000 cd m^–2^ before suffering a roll-off that rapidly accelerates beyond 10 000 cd m^–2^. They have a luminous efficiency of 25 cd A^–1^ and a luminous power efficiency of 10 lm W^–1^ at 100 cd m^–2^, rising to 37 cd A^–1^ and 13 lm W^–1^ at 1000 cd m^–2^ and peaking at 38 cd A^–1^ and 14 lm W^–1^. The CuSCN HIL/HTL devices show clearly superior performance in this display-relevant luminance range with 51 cd A^–1^ and 35 lm W^–1^ at 100 cd m^–2^ and 47 cd A^–1^ and 22 lm W^–1^ at 1000 cd m^–2^. Their efficiency roll-off is initially slow but accelerates from ≈1000 cd m^–2^ such that beyond 2000–3000 cd m^–2^ the PEDOT:PSS devices are more efficient. The performance of both device types then rapidly declines beyond 10 000 cd m^–2^.

**Table 2:** Device performance data comparison for PEDOT:PSS and CuSCN HIL/HTL containing OLED structures

HIL/HTL	Voltage [V] @ 1 cd m^–2^	Luminance [cd m^–2^] @12 V	Luminous efficiency [cd A^–1^]	Luminous power efficiency [lm W^–1^]
PEDOT	6.2	28 780	37 (@1000 cd m^–2^) 25 (@100 cd m^–2^) (38 maximum)	13 (@1000 cd m^–2^) 10 (@100 cd m^–2^) (14 maximum)
CuSCN	2.7	12 550	47 (@1000 cd m^–2^) 51 (@100 cd m^–2^) (51 maximum)	22 (@1000 cd m^–2^) 35 (@100 cd m^–2^) (55 maximum)

The roll-off in OLED device efficiency can arise from several factors but given the otherwise equivalence of our two device structures, we focus on differences between their HTL materials. Devices with steeply rising luminance–voltage characteristics tend to have better efficiency resilience.[29] This has been achieved in vacuum-processed devices by either redox doping of the HTL/ETL materials or using materials with high charge-carrier mobilities. Consequently, the fact that CuSCN is an intrinsic semiconductor with a relatively low charge-carrier mobility,[14a,b] whereas PEDOT:PSS is an oxidatively doped conductor provides a first-order explanation of the observed differences. This explanation is, moreover, supported by preliminary experiments (data not shown) in which oxidative doping of the CuSCN HIL/HTL leads to significantly improved high-voltage performance.

The detailed steps involved in forming the emissive state on the (PPy)_2_Ir(acac) dopant molecule have not yet been explored for our EML blend but it is anticipated that the majority of excitons first form as singlets on the TCTA host. Subsequent intersystem crossing, Förster transfer and Dexter transfer[18,19] generate the emissive states that undergo radiative decay. The contribution to efficiency roll-off from exciton quenching, especially at higher voltages where exciton densities rise, remains to be explored but should be very similar for both PEDOT:PSS and CuSCN HIL/HTL device types.

In terms of high luminance (>10 000 cd m^–2^), both of our device types perform less well than the highly complex multilayer vacuum-processed devices that reach 10 000 cd m^–2^ at ≈5 V.[2] Here, CuSCN HIL/HTL devices require ≈10.7 V to achieve 10 000 cd m^–2^ and PEDOT:PSS devices require ≈9.7 V. Achieving a similar performance for simple (low layer count), solution-processed OLEDs to that of the best vacuum-processed structures remains a major challenge but further optimization of CuSCN as an HIL/HTL looks a promising direction of travel.

In summary, we have demonstrated the suitability of CuSCN as a new HIL/HTL for solution-processed OLEDs. Phosphorescent green (PPy)_2_Ir(acac)-based EML blend devices with a CuSCN HIL/HTL show superior performance at display-relevant luminance relative to conventional PEDOT:PSS HIL/HTL devices, especially in respect of (i) low leakage currents below the forward bias turn-on and for reverse biases, (ii) a low turn-on voltage, and (iii) a higher luminous (cd A^–1^) and luminous power (lm W^–1^) efficiency at both 100 and 1000 cd m^–2^. Their maximum luminance values exceed 10 000 cd m^–2^ and their best efficiencies to date reach ≤50 cd A^–1^ and ≤55 lm W^–1^. This performance is attributed to efficient hole-injection, electron-blocking, and good charge balance associated with the use of the CuSCN HIL/HTL. Our's is the first report to date, to our knowledge, of a relatively thick, simple to process, low-cost, and effective OLED HIL/HTL material for use as an alternative to PEDOT:PSS. We believe these results will be of significant interest to those interested in both OLED and OPV devices and in optoelectronics more generally.

## Experimental Section

*Materials and Thin Film Characterization*: The PEDOT:PSS (Clevios VP Al-4083) HIL/HTL material was purchased from Ossila and used after dilution (1:1 by volume) with ethanol. CuSCN powder was purchased from Sigma–Aldrich and used as received. The small molecule EML host hole-transport materials 26DCzPPy and TCTA, EML guest emission material (PPy)_2_Ir(acac), and ETL material B3PYMPM were purchased from Lumtec (Taiwan) and used as received. The chemical structures of these materials are shown in Figure 1c. Film thicknesses were measured using a Dektak Surface profilometer and AFM surface topography images were acquired using an Agilent 5500 system in close-contact tapping mode. Thin film optical spectra were measured with a Shimadzu UV-3100 spectrophotometer equipped with an ISR-2600Plus integrating sphere. Photoluminescence (PL) spectra were recorded with a FluoroMax-3 spectrofluorimeter.

*Device Fabrication and Characterization*: OLEDs were fabricated on glass substrates furnished with ≈150-nm thick, 20–30 Ohm/square, prepatterned ITO anode structures. The substrates were cleaned in an ultrasonic bath using, in order, acetone, soap solution, ethanol, and isopropyl alcohol and then blow dried with nitrogen gas before being subjected to oxygen plasma (Emitech K1050X plasma Asher) treatment at 90 W for 5 min to increase the ITO work function from 4.6 to 4.9 eV. Subsequently, an HIL/HTL layer was deposited on top. For PEDOT:PSS, the as-supplied Al-4083 water solution was diluted (1:1 ratio) with ethanol (boiling point 78 °C), filtered through a 0.45-μm PVDF filter, and dispensed onto the substrate before initiating a two-step spin-coating protocol. First, the solution was evenly spread and solidified during a 1000 rpm, 60 s spin and second the resulting film was dried by spinning at 2000 rpm for 60 s, and subsequently annealed on a hotplate at 120 °C for 15 min under ambient conditions. The resulting PEDOT:PSS film was ≈45-nm thickness with work function ≈4.9 eV. For CuSCN, the as-supplied powder was dissolved in DES (boiling point: 92 °C) at 20 mg ml^–1^ concentration and subjected to continuous overnight stirring. The desired ≈45-nm thickness CuSCN HIL/HTL films were prepared by spin coating this solution at 2000 rpm for 60 s and annealing at 120 °C on a hot plate for 15 min. This latter annealing step was found not to be essential but was nevertheless used in order to match the PEDOT:PSS processing conditions. In the next OLED fabrication step, the HIL/HTL-coated substrates were transferred into a nitrogen filled glovebox and the EML spin coated on top. Three small molecules, namely the host materials 26DCzPPy and TCTA and the phosphorescent emissive dopant (PPy)_2_Ir(acac), were each dissolved at 20 mg ml^–1^ concentration in separate vials of CB by continuous overnight stirring with heating at 140 °C. Aliquots of the 26DCzPPy and TCTA solutions (in a 60:40 volume ratio) were mixed in a fresh vial and 5 wt% of the (PPy)_2_Ir(acac) solution was added. The three-component EML mixture was then heated at 140 °C for 3 h prior to spin coating at 2000 rpm for 60 s. The resulting film (45–50 nm thickness) was annealed at 120 °C for 15 min. Sequential shadow-masked, vacuum (5 × 10^−6^ mbar) deposition of a 40-nm thickness B3PYMPM ETL and a bilayer 15 nm calcium (Ca) and 80 nm aluminum (Al) cathode was performed within a thermal evaporation chamber inside the glovebox. Devices on glass substrates were cross-sectioned using a FEI Helios NanoLab 600 focused ion beam. TEM images were acquired on a JEOL 2000FX TEM microscope operating at 200 kV and used both for thickness measurement of the strata in the multilayer device stack and to observe their nanostructures. Current–voltage–luminance (J–V–L) characteristics were acquired under computer control using a Keithley 2400 source measure unit and a Minolta CS-100 calibrated luminance meter (assuming Lambertian emission). OLED emission spectra were recorded with a calibrated CCD spectrometer (Ocean Optics 2000). All measurements were performed in ambient atmosphere without encapsulation. The active area for each OLED (defined by the overlap of ITO anodes and Ca/Al cathodes) was 3 mm^2^.

## Supporting Information

As a service to our authors and readers, this journal provides supporting information supplied by the authors. Such materials are peer reviewed and may be re-organized for online delivery, but are not copy-edited or typeset. Technical support issues arising from supporting information (other than missing files) should be addressed to the authors.

## References

[1a] Tang CW, Van Slyke SA (1987). Appl. Phys. Lett.

[1b] Burroughes JH, Bradley DDC, Brown AR, Marks RN, Mackay KD, Friend RH, Burn PL, Holmes AB (1990). Nature.

[2a] Reineke S, Lindner F, Schwartz G, Seidler N, Walzer K, Lussem B, Leo K (2009). Nature.

[2b] Wang ZB, Helander MG, Qiu J, Puzzo DP, Greiner MT, Hudson ZM, Wang S, Liu ZW, Lu ZH (2011). Nat. Photon.

[3a] Suzuki M, Fukagawa H, Nakajima Y, Tsuzuki T, Takei T, Yamamoto T, Tokito S (2013). J. Soc. Info. Display.

[3b] Chung D-Y, Huang J, Bradley DDC, Campbell AJ (2009). Org. Electron.

[3c] Chung D-Y, Leem D-S, Bradley DDC, Campbell AJ (2010). Appl. Phys. Lett.

[3d] Tekoglu S, Hernandez-Sosa G, Kluge E, Lemmer U, Mechau N (2011). Org. Electron.

[3e] Sandstrom A, Dam HF, Krebs FC, Edman L (2012). Nat. Commun.

[4a] Jou J-H, Yang Y-M, Chen S-Z, Tseng J-R, Peng S-H, Hsieh C-Y, Lin Y-X, Chin C-L, Shyue J-J, Sun S-S, Chen C-T, Wang C-W, Chen C-C, Lai S-H, Tung F-C (2014). Adv. Opt. Mater.

[4b] Duan L, Hou LD, Lee TW, Qiao J, Zhang DQ, Dong GF, Wang LD, Qiu Y (2013). J. Mater. Chem.

[4c] Rehmann N, Hertel D, Meerholz K, Becker H, Heun S (2010). Appl. Phys. Lett.

[4d] Cai M, Xiao T, Hellerich E, Chen Y, Shinar R, Shinar J (2007). Adv. Mater.

[4e] Chen J, Shi C, Fu Q, Zhao F, Hu Y, Feng Y, Ma D (2011). J. Mater. Chem.

[4f] Fu Q, Chen J, Shi C, Ma D (2012). ACS Appl. Mater. Interface.

[4g] Levell JW, Zhang S, Lai W-Y, Lo S-C, Burn PL, Samuel IDW (2012). Opt. Express.

[4h] Zuniga CA, Abdallah J, Haske W, Zhang Y, Coropceanu I, Barlow S, Kippelen B, Marder SR (2012). Adv. Mater.

[4i] Liu S, Liu R, Chen Y, Ho S, Kim JH, So F (2013). Chem. Mater.

[4j] Ho S, Xiang C, Liu R, Chopra N, Mathai M, So F (2014). Org. Electron.

[4k] Yook KS, Lee JY (2014). Adv. Mater.

[4l] Höfle S, Pfaff M, Do H, Bernhard C, Gerthsen D, Lemmer U, Colsmann A (2014). Org. Electron.

[4m] Earmme T, Jenekhe SA (2012). J. Mater. Chem.

[5a] Po R, Carbonera C, Bernardi A, Camaioni N (2010). Energy Environ. Sci.

[5b] Ma H, Yip H-L, Huang F, Jen AK-Y (2011). Adv. Funct. Mater.

[5c] Walzer K, Maennig B, Pfeiffer M, Leo K (2007). Chem. Rev.

[6a] Kim J-S, Granström M, Friend RH, Johansson N, Salaneck WR, Daik R, Feast WJ, Cacialli F (2005). J. Appl. Phys.

[6b] Groenendaal L, Jonas F, Freitag D, Pielartzik H, Reynolds JR (1998). Adv. Mater.

[6c] Huang J, Miller PF, Wilson JS, de Mello JC, de Mello AJ, Bradley DDC (2000). Adv. Funct. Mater.

[6d] Kim Y, Ballantyne AM, Nelson J, Bradley DDC (2009). Org. Electron.

[7a] Jonas F, Krafft W, Muys B (2004). Macromol. Symp.

[7b] Groenendaal L, Jonas F, Freitag D, Pielartzik H, Reynolds JR (1995). Adv. Mater.

[7c] Nguyen TP, de Vos SA (2000). Appl. Surf. Sci.

[7d] de Jong MP, van IJzendoorn LJ, de Voigt MJA (2000). Appl. Phys. Lett.

[8a] Jin R, Levermore PA, Huang J, Wang X, Bradley DDC, DeMello JC (2010). Phys. Chem. Chem. Phys.

[8b] Roberts M, Asada K, Cass M, Coward C, King S, Lee A, Pintani M, Ramon M, Foden C (2009). Proc. SPIE.

[8c] Bailey J, Wright EN, Wang X, Walker AB, Bradley DDC, Kim J-S (2014). J. Appl. Phys.

[9a] Höfle S, Bruns M, Strässle S, Feldmann C, Lemmer U, Colsmann A (2007). Adv. Mater.

[9b] Girotto C, Voroshazi E, Cheyns D, Heremans P, Rand BP (2013). ACS Appl. Mater. Interface.

[9c] Manders JR, Tsang SW, Hartel MJ, Lai TH, Chen S, Amb CM, So F (2011). Adv. Funct. Mater.

[9d] Meyer J, Hamwi S, Bülow T, Johannes HH, Riedl T, Kowalsky W (2013). Appl. Phys. Lett.

[9e] Matsushima T, Jin G-H, Murata H (2007). J. Appl. Phys.

[9f] You H, Dai Y, Zhang Z, Ma D (2008). J. Appl. Phys.

[9g] Greiner MT, Helander MG, Tang W, Wang Z, Qiu J, Lu Z (2012). Nat. Mater.

[10] Greiner MT, Chai L, Helander MG, Tang WM, Lu ZH (2012). Adv. Funct. Mater.

[11a] Chen S, Manders JR, Tsang SW, So F (2012). J. Mater. Chem.

[11b] Stubhan T, Li N, Luechinger NA, Halim SC, Matt GJ, Brabec CJ (2012). Adv. Energy Mater.

[11c] Zilberberg K, Meyer J, Riedl T (2012). J. Mater. Chem. C.

[11d] Greiner MT, Lu ZH (2013). NPG Asia Mater.

[11e] Steim R, Kogler FR, Brabec CJ (2013). J. Mater. Chem.

[11f] Yip HL, Jen AKY (2010). Energy Environ. Sci.

[11g] Ma H, Yip HL, Huang F, Jen AKY (2010). Adv. Funct. Mater.

[12] Youn JH, Baek SJ, Kim HP, Nam DH, Lee Y, Lee JG, Jang J (2013). J. Mater. Chem. C.

[13] Jaffe JE, Kaspar TC, Droubay TC, Varga T, Bowden ME, Exarhos GJ (2010). J. Phys. Chem. C.

[14a] Pattanasattayavong P, Yaacobi-Gross N, Zhao K, Ndjawa GON, Li J, Yan F, O'Regan BC, Amassian A, Anthopoulos TD (2013). Adv. Mater.

[14b] Pattanasattayavong P, Yaacobi-Gross N, Zhao K, Ndjawa GON, Li J, Yan F, O'Regan BC, Amassian A, Anthopoulos TD (2013). Chem. Commun.

[15] Yaacobi-Gross N, Treat ND, Pattanasattayavong P, Faber HA, Perumal A, Stingelin N, Bradley DDC, Stavrinou PN, Heeney M, Anthopoulos TD (2014). Adv. Energy Mater.

[16] Kim SY, Jeong WI, Mayr C, Park YS, Kim KH, Lee JH, Kim JJ (2013). Adv. Funct. Mater.

[17a] Kawamura Y, Goushi K, Brooks J, Brown JJ, Sasabe H, Adachi C (2005). Appl. Phys. Lett.

[17b] Kawamura Y, Sasabe H, Adachi C (2004). Jap. J. Appl. Phys.

[18a] Baldo MA, Forrest SR (2000). Phys. Rev. B.

[18b] Baldo MA, Adachi C, Forrest SR (2000). Phys. Rev. B.

[19] Baldo MA, O'Brien DF, You Y, Shoustikov A, Sibley S, Thompson ME, Forrest SR (1998). Nature.

[20a] Su S-J, Gonmori E, Sasabe H, Kido J (1994). Adv. Mater.

[20b] Kuwabara Y, Ogawa H, Inada H, Noma N, Shirota Y (2008). Adv. Mater.

[20c] Su S-J, Sasabe H, Takeda T, Kido J (2008). Chem. Mater.

[21] Sasabe H, Chiba T, Su S-J, Pu Y-J, Nakayama K, Kido J (2008). Chem. Commun.

[22a] Lamansky S, Djurovich P, Murphy D, Adbel-Razzaq F, Lee H-E, Adachi C, Burrows PE, Forrest SR, Thompson ME (2001). J. Am. Chem. Soc.

[22b] Tsuzuki T, Tokito S (2007). Adv. Mater.

[23] Agostinelli T, Liliu S, Labram JG, Campoy-Quiles M, Hampton M, Pires E, Rawle J, Bradley DDC, Anthopoulos TD, Nelson J, MacDonald JE (2011). Adv. Funct. Mater.

[24a] Koh TW, Choi JM, Lee S, Yoo S (2013). Adv. Mater.

[24b] Löser F, Romainczyk T, Rothe C, Pavicic D, Haldi A, Hofmann M, Birnstock J (2010). J. Photonic Energy.

[24c] Chang HW, Lee J, Hofmann S, Kim YH, Müller-Meskamp L, Lüssem B, Leo K, Gather MC (2012). J. Appl. Phys.

[24d] Chen S, Kwok HS (2014). Israel J. Chem.

[25a] Rau U, Paetzold UW, Kirchartz T (2014). Phys. Rev. B.

[25b] Paci B, Bailo D, Albertini VR, Wright J, Ferrero C, Spyropoulos GD, Kymakis E (2013). Adv. Mater.

[26] Kéna-Cohen S, Stavrinou PN, Bradley DDC, Maier SA (2011). Appl. Phys. Lett.

[27a] Ratcliff EL, Bakus RC, Welch GC, van der Poll TS, Garcia A, Cowan SR, MacLeod BA, Ginley DS, Bazan GC, Olson DC (2013). J. Mater. Chem. C.

[27b] Poplavskyy D, Nelson J, Bradley DDC (2003). Appl. Phys. Lett.

[28] Khodabakhsh S, Poplavskyy D, Heutz S, Nelson J, Bradley DDC, Murata H, Jones TS (2004). Adv. Funct. Mater.

[29] Murawski C, Leo K, Gather M (2013). Adv. Mater.

