# Supplementary material for: High-Efficiency, Solution-Processed, Multilayer Phosphorescent Organic Light-Emitting Diodes with a Copper Thiocyanate Hole-Injection/Hole-Transport Layer
Source: Adv Mater. 2014 Nov 10;27(1):93–100. doi: 10.1002/adma.201403914 (PMC4315901; doi:10.1002/adma.201403914)
Supplement: Supplementary file 1 — Supplementary [file adma0027-0093-sd1.pdf]

Copyright WILEY-VCH Verlag GmbH & Co. KGaA, 69469 Weinheim, Germany, 2014.

ADVANCED MATERIALS

Supporting Information

for *Adv. Mater.,* DOI: 10.1002/adma.201403914

High-Efficiency, Solution-Processed, Multilayer Phosphorescent Organic Light-Emitting Diodes with a Copper Thiocyanate Hole-Injection/Hole-Transport Layer

Ajay Perumal, Hendrik Faber, Nir Yaacobi-Gross, Pichaya Pattanasattayavong, Claire Burgess, Shrawan Jha, Martyn A. McLachlan, Paul N. Stavrinou, Thomas D. Anthopoulos,* and Donal D. C. Bradley**

Copyright WILEY-VCH Verlag GmbH & Co. KGaA, 69469 Weinheim, Germany, 2013.

Supporting Information

High-efficiency, solution-processed, multilayer phosphorescent organic light emitting diodes with a copper thiocyanate hole injection/transport layer

Ajay Perumal,* Hendrik Faber, Nir Yaacobi-Gross, Pichaya Pattanasattayavong, Claire Burgess, Shrawan Jha, Martyn McLachlan, Paul N. Stavrinou, Thomas D. Anthopoulos* and Donal D.C. Bradley*

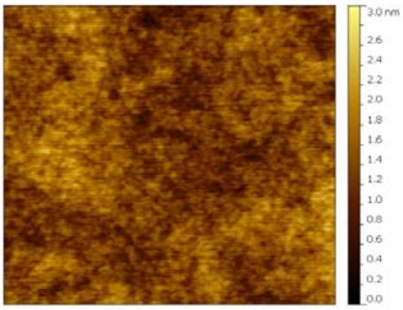

Figure S1. AFM image of the EML blend spin-coated on a quartz substrate. The root mean square (RMS) roughness is 0.32 nm. The EML blend comprised a guest-host system with 5 wt % of the guest phosphorescent green emitter bis(2-phenylpyridine)iridium(III) ($(PPy)_2Ir(acac)$) dispersed in a 60:40 volume ratio host blend of 2,6-bis(3-(carbazol-9-yl)phenyl)pyridine (26DCzPPy) and 4,4’,4”-tris(N-carbazolyl)triphenylamine (TCTA).

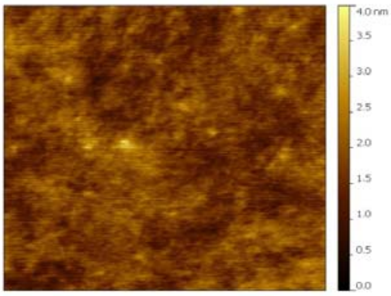

Figure S2. AFM image of the EML blend spin-coated on top of a CuSCN coated quartz substrate. The RMS roughness is 0.36 nm and the blend composition is as described in the caption to Figure S1.

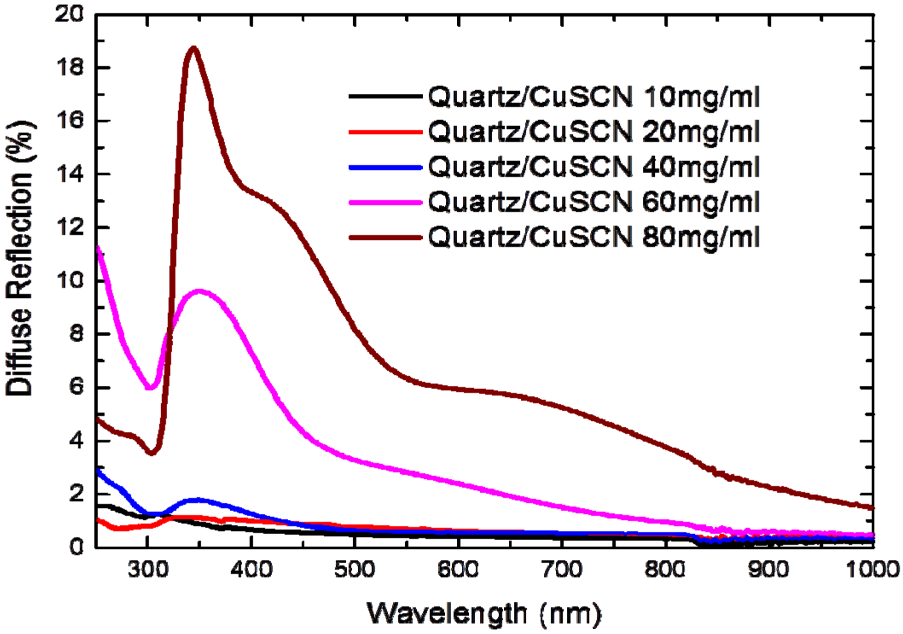

Figure S3. Diffuse reflection spectra for CuSCN films spin-coated on quartz substrates. Increasing film thicknesses were obtained by increasing the solution concentration whilst keeping the spin coating parameters constant. Scattering is seen to increase with increasing thickness. The diffuse reflection spectrum of the $BaSO_4$ integrating sphere coating is also shown for reference.
